# Assessing Hassawi Rice Straw as a Solid Biofuel: High Heating Rate Combustion Behaviour, Kinetics, and Thermodynamic Analysis

**DOI:** 10.3390/polym18131642

**Published:** 2026-07-01

**Authors:** Mohamed Anwar Ismail, Ibrahim Dubdub, Suleiman Mousa, Abdulrahman Almithn

**Affiliations:** 1 Mechanical Engineering Department, College of Engineering, King Faisal University, P.O. Box 380, Al-Ahsa 31982, Saudi Arabia; maismail@kfu.edu.sa; 2 Chemical Engineering Department, College of Engineering, King Faisal University, P.O. Box 380, Al-Ahsa 31982, Saudi Arabia; saamousa@kfu.edu.sa (S.M.); aalmithn@kfu.edu.sa (A.A.)

**Keywords:** Hassawi rice straw, combustion, TGA kinetics, thermodynamic parameters, solid biofuel, bioenergy, activation energy, lignocellulosic waste

## Abstract

This study investigated the combustion behaviour of Hassawi rice straw (HRS) at industrially relevant high heating rates through a combination of detailed physicochemical characterisation and non-isothermal thermogravimetric analysis. The biomass was characterised for proximate and ultimate composition, lignocellulosic fibre fractions (Van Soest method), and surface functional groups (FTIR). Thermogravimetric combustion experiments were conducted at heating rates of 20, 40, 60, and 80 K min^−1^ under oxidative conditions. The results demonstrate that HRS is a promising renewable solid biofuel, with high volatile matter content (72.48 wt%), moderate ash (10.27 wt%), and a higher heating value of 16.04 MJ kg^−1^. Ultimate analysis revealed low nitrogen (0.67 wt%) and sulphur (0.31 wt%) levels, indicating low potential for NOx and SOx emissions. Thermal decomposition proceeded through three distinct stages, with the main devolatilisation phase occurring between 515 and 680 K due to the breakdown of hemicellulose and cellulose. Kinetic evaluation using six model-free isoconversional methods (FR, FWO, KAS, STK, K, and VY) together with the Coats–Redfern model-fitting approach yielded an average apparent activation energy of 139 kJ mol^−1^, with the three-dimensional diffusion (D3) model providing the best fit mechanism to the experimental data. Thermodynamic analysis showed positive ΔH and ΔG values with predominantly negative ΔS, confirming the endothermic and non-spontaneous character of the process. These findings offer valuable kinetic and thermodynamic parameters for the design of efficient combustion systems utilising Hassawi rice straw as a sustainable biofuel in arid regions.

## 1. Introduction

The escalating global demand for energy, driven by rapid industrialisation and population growth, has accelerated the depletion of conventional fossil fuels and increased greenhouse gas emissions (IEA, 2025) [1]. To mitigate climate change and address the impending energy crisis, the transition toward renewable and carbon-neutral energy sources has become a global imperative. Biomass, derived from agricultural residues, forest waste, and dedicated energy crops, has emerged as one of the most promising renewable resources due to its wide availability and carbon-neutral characteristics (Basu, 2018; FAO, 2025) [2,3]. Currently, bioenergy ranks as the fourth largest global energy source, with direct combustion being the most established thermochemical conversion pathway for power generation [1,4].

Rice (*Oryza sativa* L.) is the third most important staple crop worldwide, generating enormous quantities of agricultural residue. The annual global production of rice straw is estimated between 740 and 1111 million tons (Sakhiya et al., 2021; Singh et al., 2021) [5,6]. Rice straw is recognised as one of the most abundant lignocellulosic biomass resources. Its structural matrix constitutes a complex natural polymer blend, typically composed of intertwined biopolymers including cellulose (35–40%), hemicellulose (17–25%), and lignin (10–20%), alongside silica (8–15%) and various extractives (Calvo et al., 2004; Kongkaew et al., 2015; Lin et al., 2025) [7,8,9]. The thermal degradation behaviour of these macromolecular components ultimately dictates the overall combustion kinetics. In the Eastern Province of Saudi Arabia, particularly in the Al-Ahsa Oasis, the locally adapted Hassawi rice variety produces substantial amounts of rice. Much of this residue remains underutilised or is openly burned, contributing to air pollution while representing a missed opportunity for sustainable energy production (Abd-Rabboh et al., 2022) [10].

Extensive research has investigated the thermal decomposition and kinetic behaviour of rice straw using thermogravimetric analysis (TGA). Foundational studies by Calvo et al. (2004) [7] examined heating characteristics and kinetics in different atmospheres, while later works explored co-combustion with paper sludge (Xie and Ma, 2013) [11], oxy-fuel conditions (Tang et al., 2018) [12], and co-pyrolysis (Kai et al., 2017) [13]. Recent studies have applied advanced isoconversional and model-fitting methods to rice straw from various regions (Sakhiya et al., 2021; Singh et al., 2021; Mehdi Hassan et al., 2024; Kumar et al., 2025; Lin et al., 2025) [5,6,9,14,15].

However, a critical gap persists: the vast majority of existing rice straw studies, including those on Hassawi rice, have been conducted at relatively low laboratory-scale heating rates (≤20 K min^−1^) under inert or limited oxidative conditions [5,6,7,8,11,12,14,16]. These slow-heating experiments do not adequately replicate the rapid thermal gradients, fast devolatilisation, and overlapping reaction stages encountered in industrial combustion systems such as fluidised-bed boilers. As kinetic parameters and reaction mechanisms are highly sensitive to heating rate, this limitation restricts the practical applicability of the current data for process design and scale-up.

Building upon our group’s established methodological framework for high-heating-rate combustion of agricultural wastes, previously applied to orange peel (Mousa et al., 2025), mango peel (Ismail et al., 2025a), lemon peel (Ismail et al., 2025b) [17,18,19], and peanut shell (Mousa et al., 2026) [20], the present study addresses this gap by investigating Hassawi rice straw combustion at industrially relevant heating rates of 20, 40, 60, and 80 K min^−1^. Industrial systems, such as fluidised-bed combustors (FBCs), subject biomass particles to rapid thermal gradients where devolatilisation is practically instantaneous and reaction stages overlap (Basu, 2018; Dasgupta and Das, 2017) [2,21]. While conventional TGA at <20 K min^−1^ minimises thermal lag for ideal kinetic modelling, it fails to capture the mass and heat transport limitations that dominate under these industrial conditions. The primary objectives are: (1) to perform a comprehensive physicochemical and morphological characterisation (proximate/ultimate analysis, fibre composition, HHV, FTIR, XRD, and SEM); (2) to elucidate its combustion behaviour using TGA; (3) to determine kinetic parameters using six model-free isoconversional methods, FR, FWO, KAS, STK, K, and VY, together with the Coats–Redfern (CR) model-fitting method; and (4) to evaluate thermodynamic parameters (ΔH, ΔG, and ΔS) to assess reaction feasibility and spontaneity. This work provides the first detailed high-heating-rate kinetic and thermodynamic dataset for Hassawi rice straw, offering essential insights for its sustainable valorisation as a solid biofuel in Saudi Arabia and similar arid-region contexts.

## 2. Materials and Methods

### 2.1. Feedstock Collection and Sample Preparation

In this study, Hassawi rice straw (HRS) was collected from local farms in the Al-Ahsa Oasis, Eastern Province, Saudi Arabia. Sample preparation followed the standardised protocol established in our previous high-heating-rate combustion studies on agricultural residues [17]. Briefly, the raw straw was washed with deionised water to remove surface contaminants, manually screened, dried at 310 K for 24 h in a Memmert UN110 oven (Memmert GmbH + Co.KG, Schwabach, Germany), and milled using an IKA MF 10 Basic laboratory mill (IKA-Werke GmbH & Co. KG, Staufen, Germany), followed by an ELE International shaker (ELE International, Milton Keynes, UK). A mechanical sieving analysis (Figure S1, Supplementary Materials) determined that the bulk average particle size was 0.285 mm, with the maximum yield (40.3%) retained around the 0.3 mm mesh. To eliminate mass and heat transfer variations during thermal analysis while maintaining a highly representative sample, oversized particles and ultra-fine dust were discarded to strictly isolate this dominant 0.285 ± 0.05 mm fraction. This fraction prevents the severe thermal gradients seen in large particles while avoiding the bed agglomeration issues sometimes seen with ultra-fine powders.

### 2.2. Physicochemical Characterisation

#### 2.2.1. Proximate and Ultimate Composition

Proximate analysis (moisture, volatile matter, and ash content) was performed using a Mettler Toledo TGA-2 Star System (Mettler-Toledo, Greifensee, Switzerland), aligning with the principles of standard thermogravimetric compositional analysis (ASTM E1131) [22]. Approximately 10 mg of sample was used. The heating program consisted of three stages: heating to 383 K under nitrogen to measure moisture, ramping to 1173 K under nitrogen to determine volatile matter, and finally holding at 823 K under oxygen to quantify ash. Fixed carbon was calculated by difference. Ultimate analysis (C, H, N, S) was carried out on a Vario EL III CHNS elemental analyser (Elementar Analysensysteme GmbH, Langenselbold, Germany), in accordance with standard test methods for agricultural biomass (ASTM D5373) [23], with oxygen determined by difference.

#### 2.2.2. Lignocellulosic Fibre Fractionation

Fibre components were quantified according to the Van Soest procedure [24] with an ANKOM 2000 automated fibre analyser (ANKOM Technology, Macedon, NY, USA). A 0.5 g portion of the straw underwent successive digestions to separate neutral detergent fibre (NDF), acid detergent fibre (ADF), and acid detergent lignin (ADL) using 72% sulphuric acid. Hemicellulose and cellulose percentages were derived from the mass differences between the extracted fractions.

#### 2.2.3. Calorific Value Determination

The higher heating value (HHV) of the biomass was measured using a Parr Model 1341EE isoperibol oxygen bomb calorimeter (Parr Instrument Company, Moline, IL, USA). The determination of the calorific value was conducted on a dry basis (0% moisture) following standard procedures outlined in ASTM D5865 [25]. Biomass pellets weighing 0.5 g were combusted inside the vessel under 3 MPa of pressurised oxygen. Benzoic acid served as the calibration standard for the apparatus, and all measurements were repeated in triplicate to ensure data reproducibility.

### 2.3. Spectroscopic and Microstructural Characterisation

#### 2.3.1. FTIR Spectral Analysis

Functional groups were identified using a JASCO FTIR-4100 spectrometer (JASCO Corporation, Tokyo, Japan). Pellets were prepared by mixing 2 mg of HRS powder with 200 mg of spectroscopic-grade KBr and pressing the mixture into transparent discs. Infrared spectra were recorded between 4000 and 400 cm^−1^ at 4 cm^−1^ resolution, collecting 32 scans per sample after background correction.

#### 2.3.2. SEM Morphological Examination

Particle morphology was observed with a JEOL JSM-7600F field-emission scanning electron microscope (JEOL Ltd., Tokyo, Japan). Samples were affixed to aluminium stubs with conductive carbon tape, coated with a thin gold layer, and imaged using the secondary electron detector at a 15.0 kV accelerating voltage and 8.0 mm working distance. Sample preparation and imaging were conducted following standard operating procedures consistent with guidelines for scanning electron microscopy (ASTM E986) [26].

### 2.4. Non-Isothermal Combustion Experiments

Combustion behaviour was investigated on a NETZSCH STA 449 F3 Jupiter simultaneous thermal analyser (NETZSCH-Gerätebau GmbH, Selb, Germany), with the experimental design and parameters established in accordance with the guidelines of the ICTAC Kinetics Committee [27]. Approximately 10 mg of HRS was placed in an alumina crucible and heated from 303 K to 1173 K under synthetic air (20% O_2_ + 80% N_2_) flowing at 60 mL min^−1^. Experiments were performed at four different heating rates (20, 40, 60, and 80 K min^−1^). The instrument’s PID controller effectively controlled the temperature during the exothermic ignition phase. All TG and DTG experiments were recorded in duplicate to confirm reproducibility. The curves presented in this study represent the average values of these replicate runs. The maximum deviation between replicates demonstrated high consistency, showing a variance of less than ±1.5% for mass loss and less than ±2 K for characteristic peak temperatures. To mitigate the risk of sample ignition and uncontrolled exothermic runaway typical at these higher heating rates, very small sample masses were utilised. This, combined with the instrument’s responsive PID controller and continuous carrier gas flow, ensured the effective dissipation of evolved heat and strict adherence to the programmed linear heating rates.

### 2.5. Kinetic Modelling and Thermodynamic Analysis

The non-isothermal combustion kinetics of the HRS were analysed using the foundational Arrhenius equation, correlating the reaction rate with absolute temperature (T) and the degree of conversion (α). The conversion fraction was extracted directly from the empirical TG curves. Aligning with the recommendations of the ICTAC Kinetics Committee [27], the apparent activation energy (*E_a_*) was determined across the conversion spectrum utilising six distinct isoconversional models. This cross-validation approach, employing one differential method (Friedman) and five integral methods (FWO, KAS, Starink, Kissinger, and Vyazovkin) (Table S1, Supplementary Materials), was selected to ensure robust estimation of the kinetic triplet and eliminate systemic mathematical errors inherent in assuming a single approximation.

Following *E_a_* determination, the Coats–Redfern multivariate non-linear regression technique was applied to identify the most probable solid-state reaction mechanism (f(α)) by evaluating fifteen established kinetic models, encompassing diffusion, nucleation, and geometrical contraction pathways (Table S2, Supplementary Materials) (Coats & Redfern, 1964) [28]. Standard thermodynamic parameters, including the change in enthalpy (ΔH), Gibbs free energy (ΔG), and entropy (ΔS), were calculated to determine the thermal feasibility, energy barrier, and process spontaneity of the HRS combustion. Detailed mathematical derivations and reaction model formulas are provided in the Supplementary Materials (Table S3, Equations (S11)–(S13), Supplementary Materials).

## 3. Results and Discussion

### 3.1. Physicochemical Properties of Hassawi Rice Straw

The physicochemical profile of Hassawi rice straw (HRS), including proximate, ultimate, and lignocellulosic fibre analyses, is summarised in Table 1. The proximate analysis revealed a high volatile matter content of 72.48 ± 0.32 wt%, which is comparable to the values previously reported for lemon peel (73.20 wt%) (Ismail et al., 2025b) [19] and mango peel (70.64 wt%) (Ismail et al., 2025a) [18], and slightly higher than that of peanut shell (65.30 wt%) (Mousa et al., 2026) [20]. This elevated volatile content is favourable for rapid ignition and efficient combustion in the gas-phase stage. The moisture content of 7.41 ± 0.11 wt% was well below the critical threshold of 10 wt% recommended for the stable storage of solid biofuels, thereby minimising the risk of microbial degradation (Jensen et al., 2006) [29]. The ash content (10.27 ± 0.18 wt%) was higher than that observed for orange peel (5.50 wt%) (Mousa et al., 2025) [17], mango peel (7.55 wt%) (Ismail et al., 2025a) [18], and peanut shell (6.59 wt%) (Mousa et al., 2026) [20]. This moderately elevated ash level, typical of rice straw, necessitates the careful consideration of potential slagging and fouling issues in industrial boiler applications [2,30].

The ultimate analysis (dry basis) showed a carbon content of 41.68 ± 0.18 wt% and an oxygen content of 50.06 ± 0.21 wt%. Notably, the nitrogen (0.67 ± 0.03 wt%) and sulphur (0.31 ± 0.02 wt%) contents were very low, indicating minimal potential for NOx and SOx emissions during combustion. The measured higher heating value (HHV) of 16.04 ± 0.25 MJ kg^−1^ (determined on a dry basis, 0% moisture) is consistent with the literature values reported for rice straw, which typically range from approximately 12 to 19 MJ kg^−1^ depending on ash content and origin (Zhang et al., 2018) [30]. This value is comparable to recent reports such as that by Tsai et al. (2023) [31], who measured 16.94 ± 0.21 MJ kg^−1^ for rice straw with a similar proximate composition (ash 9.48 wt%, volatile matter 74.29 wt%). Slightly lower values have also been reported for raw rice straw (e.g., 14.24 MJ kg^−1^) in other studies (Ibitoye et al., 2025) [32]. The HHV remains competitive with other agricultural residues, although it was lower than the values obtained for mango peel (21.9 MJ kg^−1^) and peanut shell (20.87 MJ kg^−1^) in our previous studies.

The lignocellulosic fibre composition of HRS consisted of 36.82 ± 0.31% cellulose, 22.64 ± 0.24% hemicellulose, 11.47 ± 0.18% lignin, and 29.07 ± 0.27% extractives. These values fall well within the typical ranges reported for rice straw (cellulose 32–42%, hemicellulose 19–27%, lignin 10–20%). The moderately high extractives fraction is consistent with the elevated silica and ash content characteristic of rice straw, particularly the Hassawi variety grown in the Al-Ahsa region (Abd-Rabboh et al., 2022) [10].

### 3.2. Structural and Surface Characterisation

#### 3.2.1. FTIR Analysis of Functional Groups

**Figure 1:**
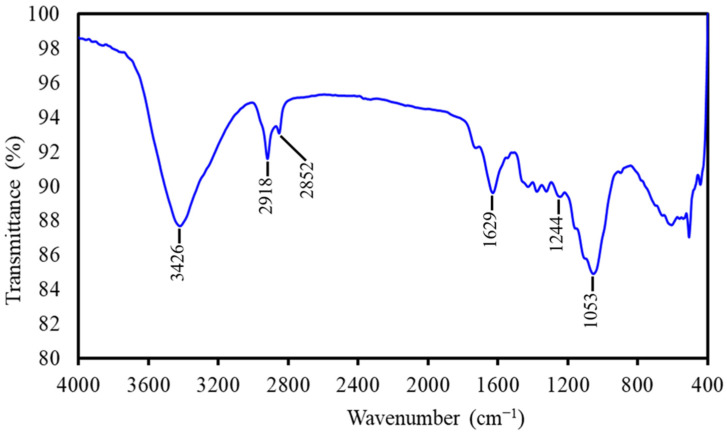
FTIR spectrum of Hassawi rice straw powder highlighting characteristic lignocellulosic and silica-related absorption bands.

The surface functional groups of Hassawi rice straw were identified using FTIR spectroscopy. The spectrum (Figure 1) displayed characteristic bands of a typical lignocellulosic biomass. The broad and intense absorption band at 3418 cm^−1^ was attributed to O–H stretching vibrations from hydroxyl groups in cellulose, hemicellulose, lignin, and adsorbed moisture. The peaks at 2918 cm^−1^ and 2852 cm^−1^ corresponded to asymmetric and symmetric C–H stretching vibrations of aliphatic methylene and methyl groups, respectively.

The band at 1629 cm^−1^ arises from C=C aromatic skeletal vibrations of lignin and/or O–H bending of absorbed water. Additional bands in the 1400–1300 cm^−1^ region are assigned to C–H bending and C–O stretching modes, while the peak at 1244 cm^−1^ is characteristic of guaiacyl ring breathing in lignin. The strongest band at 1053 cm^−1^ was dominated by C–O and C–O–C stretching vibrations of cellulose and hemicellulose, overlapping with Si–O stretching from the high silica content typical of rice straw. Lower wavenumber bands are associated with skeletal vibrations and minor mineral phases. Overall, the FTIR spectrum confirms the presence of the major lignocellulosic components and is consistent with previous reports on rice straw (Sakhiya et al., 2021; Lin et al., 2025) [5,9].

#### 3.2.2. X-Ray Diffraction and Mineralogy

**Figure 2:**
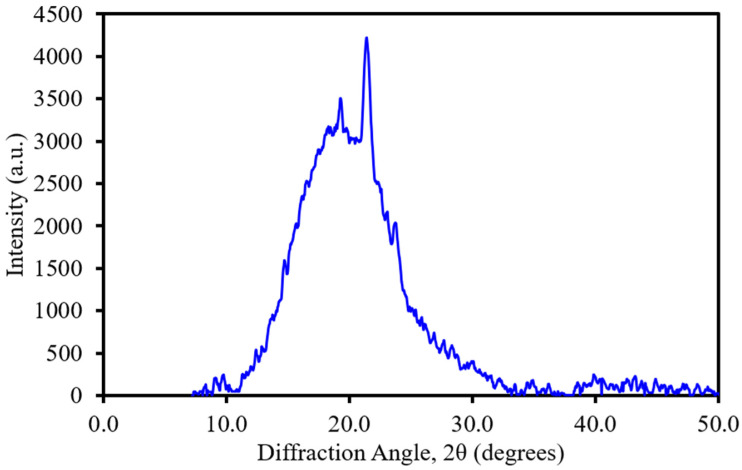
X-ray diffraction pattern of Hassawi rice straw showing the dominant amorphous halo and minor crystalline silica phases.

The crystalline structure of Hassawi rice straw was examined by XRD. The diffractogram (Figure 2) exhibited a broad, intense halo centred around 22° 2θ, which is characteristic of amorphous cellulose and amorphous silica, the dominant mineral component in rice straw. Minor sharp peaks indicate the presence of small amounts of crystalline silica phases (e.g., cristobalite). The low overall crystallinity is typical for raw rice straw and reflects the disordered arrangement of the lignocellulosic matrix combined with the high amorphous silica content (Abd-Rabboh et al., 2022) [10]. This amorphous nature facilitates thermal decomposition and is consistent with the high volatile matter content observed in the proximate analysis.

#### 3.2.3. SEM Microstructural Analysis

**Figure 3:**
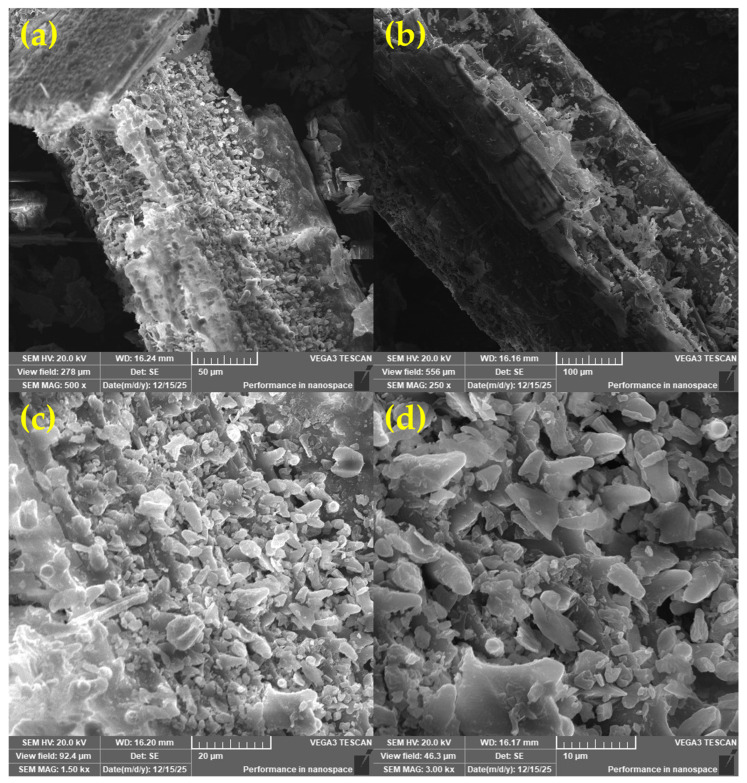
SEM images of ground Hassawi rice straw at increasing magnifications: (**a**) 500× overview, (**b**) 250× fibrous structure, (**c**) 1.50 k× surface porosity, and (**d**) 3.00 k× detailed microstructure.

The surface morphology and microstructural features of the Hassawi rice straw powder were examined using scanning electron microscopy to understand the physical characteristics that influence combustion behaviour. As shown in Figure 3, the material exhibits a highly heterogeneous and porous morphology typical of lignocellulosic straw. The low-magnification overview (Figure 3a, 500×) revealed elongated, irregular particles with rough surfaces resulting from the mechanical grinding process. At medium magnification (Figure 3b, 250×), distinct fibrous bundles and layered structures became evident, confirming the presence of cellulose and hemicellulose fibres embedded in a lignocellulosic matrix.

Closer inspection at higher magnifications (Figure 3c, 1.50 k× and Figure 3d, 3.00 k×) highlighted significant surface roughness, irregular flakes, and a well-developed network of pores and cavities. This porous microstructure is particularly important for combustion applications, as it facilitates oxygen diffusion into the particle interior and the release of volatile gases during thermal decomposition. Such features strongly support the kinetic findings (Section 3.4), where the three-dimensional diffusion (D3) model was identified as the dominant reaction mechanism. The observed morphology is consistent with previous reports on rice straw and aligns with the high extractives and silica content characteristic of the Hassawi variety grown in the Al-Ahsa region.

### 3.3. TGA Combustion Profiles

**Figure 4:**
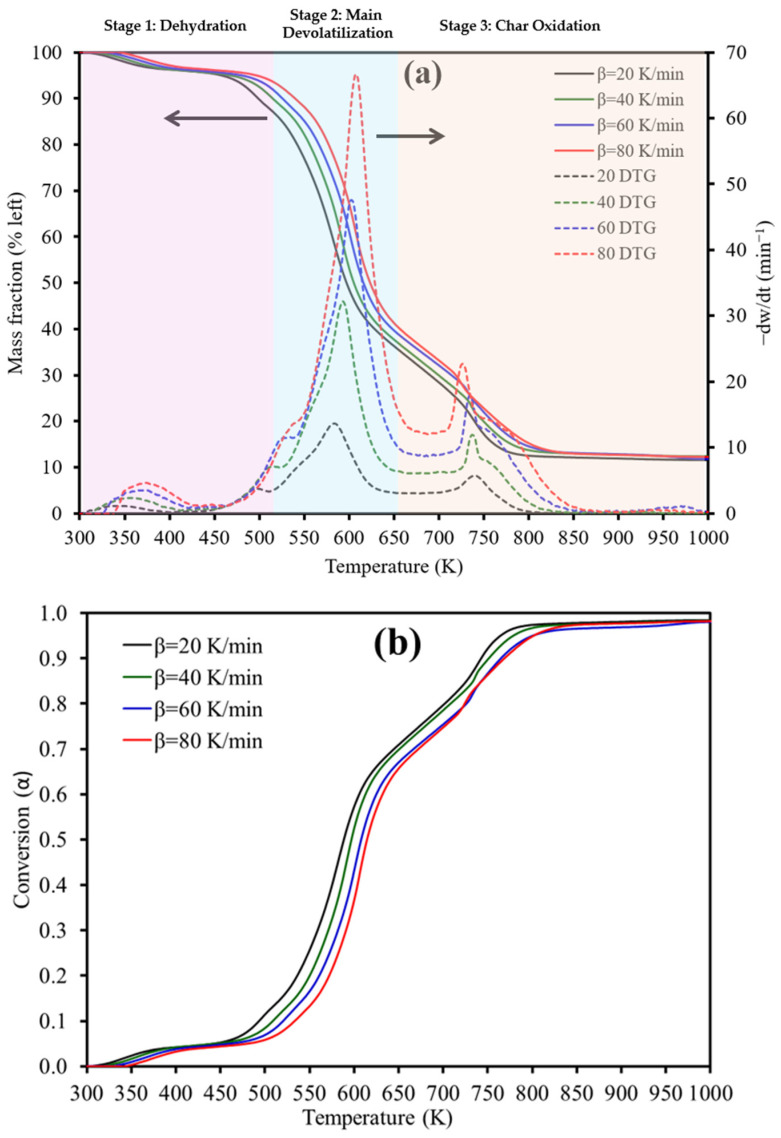
Thermal TG and DTG curves (**a**) and conversion profiles (**b**) for Hassawi rice straw combustion at heating rates of 20–80 K min^−1^. The shaded regions in (**a**) designate the three primary combustion stages: dehydration (Stage 1), main devolatilisation (Stage 2), and char oxidation (Stage 3).

Hassawi rice straw possesses favourable properties as a solid biofuel, including high volatile matter content (72.48 ± 0.32 wt%) and suitable lignocellulosic composition. The thermogravimetric (TG) and derivative thermogravimetric (DTG) profiles obtained during combustion at heating rates of 20, 40, 60, and 80 K min^−1^ are shown in Figure 4. As commonly observed in biomass thermal analysis (e.g., Tang et al., 2018 [12]; Calvo et al., 2004 [7]), increasing the heating rate produced a consistent shift of both TG and DTG curves toward higher temperatures. This behaviour results from thermal lag, whereby the limited residence time at faster heating rates prevents the particle interior from reaching immediate thermal equilibrium with the furnace, thereby delaying the onset of decomposition. Furthermore, when compared to conventional TGA studies conducted at lower heating rates (e.g., 5–10 K min^−1^), the overlapping of the hemicellulose and cellulose degradation stages becomes much more pronounced at these elevated rates, and the characteristic decomposition peaks are pushed to higher temperatures due to this thermal lag [12].

The TG and DTG curves indicate that HRS combustion occurs through three successive stages of mass loss, the details of which are summarised in Table 2. Stage 1 (dehydration) takes place between approximately 460 and 550 K and involves the evaporation of moisture and light volatiles, resulting in a mass loss of 8–9% at rates of 1–4 min^−1^. Stage 2 is the principal devolatilisation phase, occurring between 515 and 680 K, during which a major mass loss of 47–50% is recorded at peak rates of 14–72 min^−1^. This stage is driven by the thermal breakdown of the lignocellulosic matrix. In particular, the amorphous hemicellulose networks undergo rapid random chain scission and depolymerisation at the lower end of this temperature range. This is immediately followed by the cleavage of the highly crystalline β-(1→4)-glycosidic bonds within the cellulose biopolymer chains. The third stage involves the combustion of the heavily cross-linked phenolic structures of residual lignin and char burnout, occurring between 620 and 840 K with an average mass loss of approximately 16% and a mass loss rate of 6–16 min^−1^.

These findings are supported by previous studies on rice straw combustion (Calvo et al., 2004; Xie and Ma, 2013; Dasgupta and Das, 2017; Tang et al., 2018; Ngo and Chiang, 2021) [7,11,12,16,21]. For instance, Tang et al. (2018) [12] reported the first DTG peak at 577 K, which is close to the second peak observed in the present study (average ~585–607 K), and a second peak at 681 K, comparable to our third peak (~740–770 K). Similarly, Ngo and Chiang (2021) [16] described the first stage (moisture evaporation) occurring between 298–448 K with ~3.4% mass loss, the main devolatilisation stage (cellulose and hemicellulose decomposition) between 449–803 K with ~55.9% mass loss, and the final lignin/char oxidation stage above 803 K with ~11.2% mass loss.

This three-stage decomposition profile for HRS is similar to that previously reported for mango peel (Ismail et al., 2025a) [18] and orange peel (Mousa et al., 2025) [17], but contrasts with the four-stage profile observed for lemon peel (Ismail et al., 2025b) [19]. Above ~720 K (corresponding to α ≈ 0.7–0.8 in Figure 4b), the TG curves exhibited a slow, almost horizontal decline representing the gradual oxidation of the remaining char and fixed carbon. Kinetic calculations were performed over the full conversion range α = 0.1–0.9. However, the main kinetic parameters, averages, and thermodynamic analysis reported in this study were based on the active devolatilisation range α = 0.1–0.5 (where R^2^ > 0.93 for all methods and primary reactions dominate). Data at α > 0.5 (char oxidation stage) are shown for completeness only but exhibited lower linearity in some methods and were not used for the reported average *E_a_* or thermodynamic parameters.

The data in Table 2 reveals that the characteristic temperatures (*T_range_* and *T_peak_*) consistently shifted to higher values as the heating rate increased. This phenomenon is primarily attributed to the thermal lag effect. At elevated heating rates, heat transfer to the core of the biomass particle is delayed, causing the particle surface to heat much faster than its interior. Consequently, a higher environmental temperature is reached before the required activation energy for internal decomposition is fully supplied. Furthermore, devolatilisation occurs over a shorter time interval and at a higher thermal driving force, significantly increasing the maximum mass loss rate.

**Figure 5:**
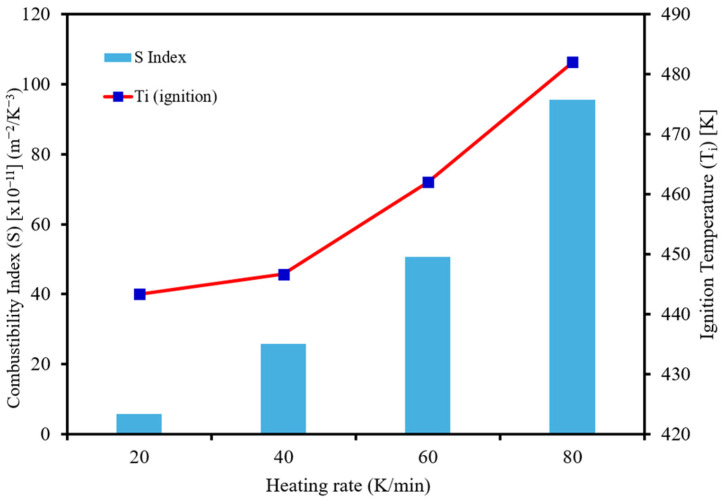
Influence of heating rate on combustibility index and ignition temperature for Hassawi rice straw.

To further quantify combustion performance, the combustibility index (*S*) was evaluated using the formula

(1) $$S=\frac{R_{max}\times R_{\mathrm{mean}}}{T_{i}^{2}\times T_{b}}$$

where $R_{max}$ and $R_{\mathrm{mean}}$ are the maximum and mean mass loss rates, and $T_{i}$ and $T_{b}$ are the ignition and burnout temperatures, respectively (Zhuikov et al., 2021) [33]. As shown in Figure 5, the combustibility index increased progressively with heating rate, rising from 6.8 × 10^−11^ K^−3^ m^−2^ at 20 K m^−1^ to 96.2 × 10^−11^ K^−3^ m^−2^ at 80 K m^−1^. This trend confirms that higher heating rates enhance the overall reactivity and oxidation intensity of HRS. Concurrently, the ignition temperature ($T_{i}$) shifts to higher values with increasing heating rate, consistent with the thermal lag effect observed in the TG/DTG curves.

### 3.4. Kinetic Analysis

#### 3.4.1. Isoconversional Kinetic Methods

**Figure 6:**
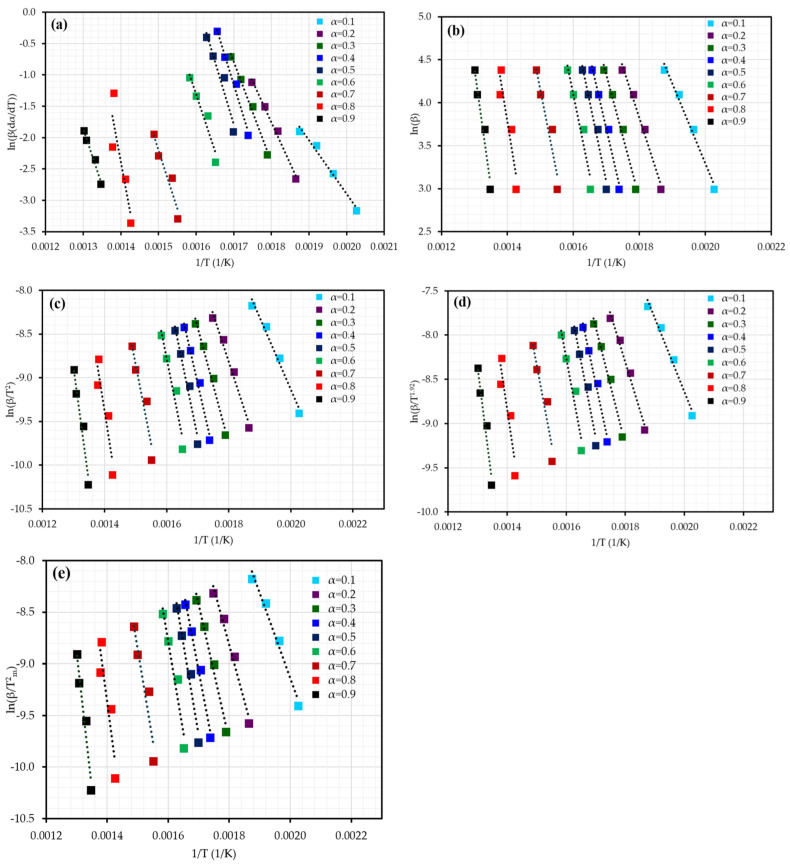
Linear fits from five isoconversional methods for Hassawi rice straw at different conversion levels: (**a**) FR, (**b**) FWO, (**c**) KAS, (**d**) STK, and (**e**) (K).

**Figure 7:**
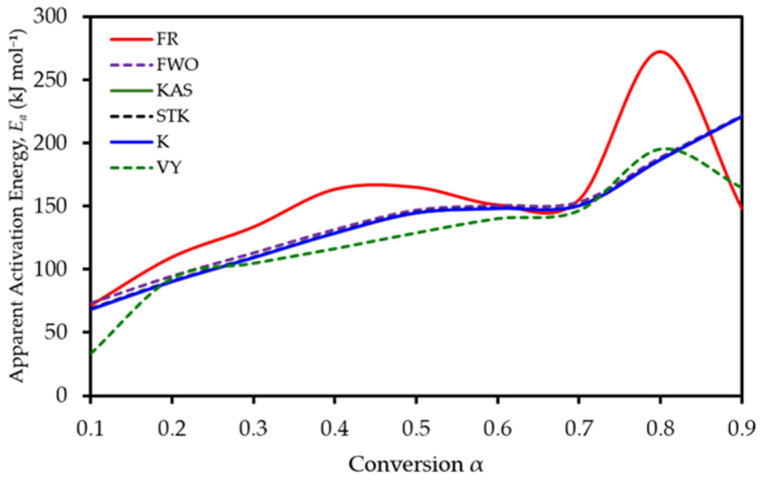
Variation of apparent activation energy with conversion for Hassawi rice straw determined by six isoconversional methods.

The apparent activation energy ($E_{a}$) of Hassawi rice straw (HRS) combustion was evaluated as a function of conversion (α) using six model-free isoconversional methods. These methods are widely endorsed by the ICTAC Kinetics Committee because they do not require any prior assumption of a specific reaction model, making them highly suitable for analysing the complex, multi-step thermal decomposition of biomass. The linear regression plots derived from the experimental TGA data for the five isoconversional methods (FR, FWO, KAS, STK, and K) are shown in Figure 6, all exhibiting high coefficients of determination ($R^{2}>0.95$) over most of the conversion range. The resulting $E_{a}$ values are listed in Table 3 (including the Vyazovkin method) and visualised in Figure 7.

The results demonstrate that HRS combustion is a multi-step process in which $E_{a}$ varies significantly with conversion. Two distinct trends were observed. For the differential FR method and the non-linear VY method, $E_{a}$ increased steadily with conversion in the ranges α=0.1–0.4 (FR) and α=0.1–0.8 (VY), with slight fluctuations at higher conversions. In contrast, the integral methods (FWO, KAS, STK, and K) showed a smoother increase up to α≈0.7, followed by a similar pattern. The FR method yielded the widest range of activation energies (71–272 kJ mol^−1^), while the VY method provided the lowest overall values (33–164 kJ mol^−1^). The four integral methods produced highly consistent results: FWO (73–222 kJ mol^−1^), KAS (68–221 kJ mol^−1^), STK (69–221 kJ mol^−1^), and K (68–221 kJ mol^−1^).

The low $E_{a}$ values observed at the beginning of conversion (33–73 kJ mol^−1^) were attributed to the breaking of weak chemical bonds in the amorphous regions and hemicellulose structures. As conversion progresses, the increase in $E_{a}$ indicates that the reaction becomes dominated by the degradation of more thermally stable crystalline cellulose and lignin components. Despite methodological differences, the average activation energy across all six methods was 139 kJ mol^−1^. These findings are consistent with previous studies on rice straw (Xie and Ma, 2013; Ngo and Chiang, 2021; Kumar et al., 2025) [11,15,16]. Xie and Ma (2013) [11] reported similar trends using the FR and FWO methods, with $E_{a}$ values ranging from 95–186 kJ mol^−1^ (FR) and 97–204 kJ mol^−1^ (FWO). Ngo and Chiang (2021) obtained an average $E_{a}$ of 89.6 kJ mol^−1^ for rice straw combustion using a multiple linearised regression approach, while Kumar et al. (2025) [15] reported pseudo-component values of 110.0 kJ mol^−1^ (hemicellulose), 142.0 kJ mol^−1^ (cellulose), and 180.9 kJ mol^−1^ (lignin) using the distributed activation energy model.

#### 3.4.2. Kinetic Compensation Effect Validation

To confirm the consistency of the kinetic parameters obtained in this study, the kinetic compensation effect (KCE) was examined. The KCE is characterised by a linear correlation between the natural logarithm of the pre-exponential factor (ln A_0_) and the apparent activation energy (E_a_), expressed by the following relationship (Zhu & Liu, 2020) [34]:

(2) $$ln\;A_{0}=a+bE_{a}$$

**Figure 8:**
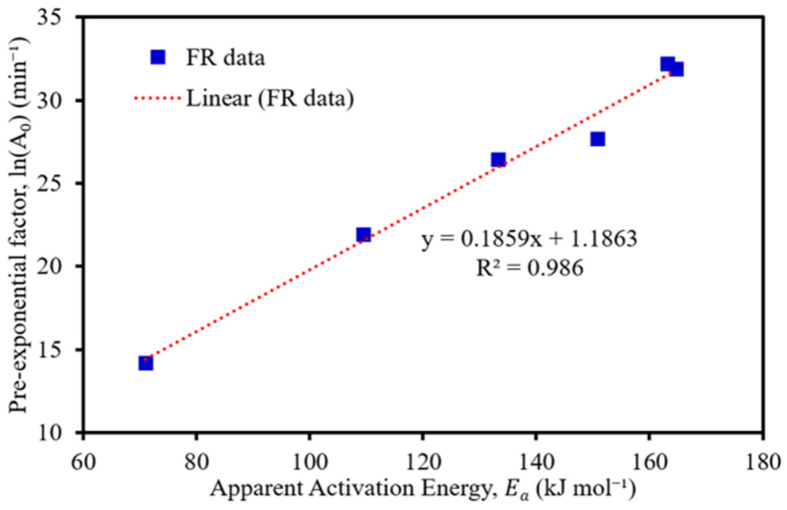
Kinetic compensation effect plot derived from the Friedman method for Hassawi rice straw combustion (α = 0.1–0.6).

Figure 8 shows the KCE plot derived from the Friedman method over the main active combustion range (α=0.1–0.6). The data exhibited strong linearity with a coefficient of determination $R^{2}=0.986$ and the regression equation y=0.1859x+1.1863. This high correlation confirms that the variations in activation energy are physically compensated by corresponding changes in the pre-exponential factor, thereby validating the consistency and robustness of the kinetic triplet determined in this study.

#### 3.4.3. Coats–Redfern Model-Fitting and Reaction Mechanism

To identify the most probable solid-state reaction mechanism governing HRS combustion, the Coats–Redfern (CR) model-fitting method was applied by screening sixteen standard solid-state reaction models (Table S4, Supplementary Materials). Because HRS combustion proceeds through three distinct stages, the CR method was applied separately to each stage (dehydration, main devolatilisation, and char oxidation). For the main active combustion stages (second and third reactions, corresponding to α ≈ 0.1–0.5), the three-dimensional diffusion model (D3, Jander equation) consistently provided the best statistical fit, yielding the highest correlation coefficients (R^2^ > 0.98). This result indicates that the oxidation rate of HRS is governed by a diffusion-controlled mechanism, with the rate expression:

(3) $$f(\alpha )=1.5{\left[1-(1-\alpha {)}^{1/3}\right]}^{-1}{\left(1-\alpha \right)}^{2/3}$$

The corresponding kinetic parameters ($E_{a}$, $\ln A_{0}$, and $R^{2}$) obtained for the D3 model at the four heating rates are presented in Table 4. This diffusion-controlled behaviour aligns with the porous microstructure observed in the SEM analysis (Figure 3), where a well-developed network of pores and cavities facilitates oxygen diffusion yet becomes rate-limiting at higher conversions. The finding is consistent with our previous studies on lemon peel and mango peel, indicating that diffusion limitations are common in high-ash lignocellulosic agro-wastes under oxidative conditions (Ismail et al., 2025a, 2025b) [18,19]. It contrasts, however, with some earlier rice straw studies that identified nucleation models as the best fit, likely due to differences in particle size or experimental heating rates (Nie et al., 2022) [35]. The identification of the D3 mechanism is further supported by Mehdi Hassan et al. (2024) [14] and Tang et al. (2018) [12], who also reported strong diffusion control during the thermal decomposition of rice straw.

Table 4 reveals distinct trends in the apparent activation energy (*E_a_*) as a function of heating rate. In Stage 2 (main devolatilisation), *E_a_* progressively increased from 105.2 to 143.4 kJ mol^−1^. This is linked to mass transfer limitations; at higher heating rates, the rapid, explosive release of volatiles creates a localised outward pressure gradient that impedes oxygen diffusion into the particle, raising the apparent energy barrier under the D3 diffusion model. Conversely, in Stage 3 (char oxidation), *E_a_* decreased steadily from 65.3 to 46.6 kJ mol^−1^. The intense devolatilisation in Stage 2 expels gases forcefully, leaving behind a highly porous, disordered char structure. This augmented porosity enhances the accessible surface area for oxygen, significantly lowering the apparent activation energy required for final burnout.

### 3.5. Thermodynamic Parameters

Thermodynamic parameters were calculated from the kinetic data obtained using the six model-free methods and the three-dimensional diffusion (D3) model. The results are summarised in Table 5. As conversion increased from α = 0.1 to 0.5, the pre-exponential factor (A_0_) showed a steady rise. This trend indicates greater molecular collision frequency as the reaction front advances through the porous structure of the biomass particles. Positive activation enthalpy (ΔH) values were obtained across the active conversion range (0.1–0.5). These results confirm that the formation of the activated complex is an endothermic process. Although the overall combustion reaction is exothermic, an energy input is still required to overcome the activation barrier. The small difference between Eₐ and ΔH (approximately 4–5 kJ mol^−1^) suggests that only modest additional energy is needed to convert the activated complex into final reaction products. Gibbs free energy (ΔG) remained positive (116–220 kJ mol^−1^) across all methods and conversion levels. This indicates that the formation of the activated complex is non-spontaneous. The higher ΔG values observed at elevated conversions point to a significant thermodynamic barrier, meaning that continuous external heat input is required to sustain the reaction, particularly during the later char oxidation stage (Sakhiya et al. 2021) [5]. The entropy change (ΔS) values were predominantly negative at low to intermediate conversions. This suggests that the activated complex possesses a higher degree of structural order than the initial reactants. Such behaviour is typical for the complex thermal degradation of lignocellulosic biomass and reflects the close connection between kinetic and thermodynamic factors during HRS combustion. The thermodynamic trends observed in this study are consistent with those reported for rice straw and similar agricultural residues under oxidative conditions (Sakhiya et al. 2021, Ismail 2025a, Ismail 2025b) [5,18,19].

### 3.6. Practical Implications for Biofuel Applications

This study provides the first comprehensive high-heating-rate combustion characterisation, kinetic modelling, and thermodynamic analysis of Hassawi rice straw (HRS) from the Al-Ahsa region of Saudi Arabia. The results confirm that HRS possesses favourable characteristics as a solid biofuel. It exhibits a high volatile matter content (72.48 wt%), moderate ash content (10.27 wt%), and a higher heating value of 16.04 MJ kg^−1^. Notably, the very low nitrogen (0.67 wt%) and sulphur (0.31 wt%) contents indicate minimal potential for NOx and SOx emissions, making HRS environmentally superior to many other agricultural residues.

When compared with rice straw from other regions, the average activation energy obtained in this study (139 kJ mol^−1^) lies within the typical range reported for rice straw combustion and pyrolysis (89–204 kJ mol^−1^) (Xie and Ma, 2013; Ngo and Chiang, 2021; Sakhiya et al., 2021; Mehdi Hassan et al., 2024; Kumar et al., 2025) [5,11,14,15,16]. The identification of the three-dimensional diffusion (D3) mechanism as rate-controlling is also consistent with several oxidative combustion studies on rice straw (Mehdi Hassan et al., 2024; Tang et al., 2018) [12,14], reinforcing that diffusion limitations through the porous char layer are common in high-ash lignocellulosic residues under industrial heating rates. Compared with the fruit peels and peanut shell previously investigated by our group, HRS shows a lower energy density but offers distinct advantages, including abundant local availability and significantly reduced emission potential.

Combustion proceeds through three distinct stages, with the main devolatilisation phase (515–680 K) dominated by hemicellulose and cellulose degradation. The combustibility index (S) increased markedly with heating rate, confirming enhanced reactivity under conditions representative of industrial combustors. From a practical perspective, HRS is well-suited for direct combustion or co-firing with coal in power plants, provided that boiler designs account for its moderate ash content and diffusion-limited kinetics. Particle size optimisation (finer grinding) is recommended to minimise diffusion limitations and improve combustion efficiency. In the context of the Al-Ahsa region, where large quantities of Hassawi rice straw are generated annually, valorisation of this residue offers a sustainable solution to agricultural waste management while contributing to Saudi Arabia’s renewable energy targets and reducing open-field burning.

## 4. Conclusions

This study presents a comprehensive physicochemical, morphological, kinetic, and thermodynamic characterisation of Hassawi rice straw (HRS) combustion at industrially relevant high heating rates (20–80 K min^−1^). The results establish HRS as a promising solid biofuel with high volatile matter content (72.48 wt%), moderate ash content (10.27 wt%), very low nitrogen (0.67 wt%) and sulphur (0.31 wt%), and a higher heating value of 16.04 MJ kg^−1^. Combustion proceeds through three distinct stages, with primary devolatilisation occurring between 515 and 680 K. Kinetic analysis using six model-free isoconversional methods yielded an average activation energy of 139 kJ mol^−1^, while the Coats–Redfern method confirmed that the reaction is governed by a three-dimensional diffusion (D3) mechanism, consistent with the porous microstructure observed via SEM. Thermodynamic parameters indicated positive ΔH and ΔG with predominantly negative ΔS, confirming the endothermic and non-spontaneous nature of the activation process. Crucially, the identification of a diffusion-limited mechanism (D3) provides physical parameters that will directly dictate the engineering design, specifically particle sizing and aerodynamic feed requirements, for scaling up HRS combustion in fluidised bed reactors. This work fills a knowledge gap by providing the first detailed high-heating-rate combustion dataset for Hassawi rice straw. The findings demonstrate good reactivity and low emission potential, offering essential insights for reactor design and process optimisation in arid-region bioenergy applications. In the Al-Ahsa region of Saudi Arabia, where large quantities of HRS are generated annually, utilisation of this residue can reduce open-field burning, mitigate air pollution, and support sustainable energy production in alignment with the strategic goals of Saudi Vision 2030. Future research should focus on pilot-scale validation of these kinetic parameters, ash slagging and fouling characteristics, co-combustion behaviour with coal, and techno-economic assessment prior to full large-scale industrial implementation.

## Figures and Tables

**Table 1:** Proximate, ultimate, and fibre composition of Hassawi rice straw (mean ± SD, *n* = 3).

Parameter	Value (wt% Dry Basis)	Parameter	Value (wt% Dry Basis)
Moisture Content	7.41 ± 0.11	Carbon (C)	41.68 ± 0.18
Volatile Matter	72.48 ± 0.32	Hydrogen (H)	7.28 ± 0.11
Ash	10.27 ± 0.18	Nitrogen (N)	0.67 ± 0.03
Fixed Carbon	9.84 ± 0.15	Sulphur (S)	0.31 ± 0.02
Hemicellulose	22.64 ± 0.24	Oxygen (O)	50.06 ± 0.21
Cellulose	36.82 ± 0.31	Lignin	11.47 ± 0.18
Extractives	29.07 ± 0.27	Heating Value	16.04 ± 0.25 MJ kg^−1^

**Table 2:** Characteristic temperatures and mass loss data for the three combustion stages of Hassawi rice straw at different heating rates.

Heating Rate (K min^−1^)	Stage 1 (Dehydration)			Stage 2 (Main Devolatilisation)			Stage 3 (Char Oxidation)		
	T_range_, T_peak_ (K)	Weight Loss %/Max Rate	Process	T_range_, T_peak_ (K)	Weight Loss %/Max Rate	Process	T_range_, T_peak_ (K)	Weight Loss %/Max Rate	Process
20	460–515, 500	8/1	Dehydration	515–620, 585	47/14	Degradation of hemicellulose and cellulose	620–790, 740	16/6	Degradation of cellulose and lignin
40	470–525, 510	8/2.5		525–640, 595	49/33		640–800, 750	16/10	
60	480–540, 518	8/3		540–675, 605	50/50		675–830, 760	16/13	
80	490–550, 537	9/4		550–680, 607	50/72		680–840, 770	16/16	

**Table 3:** Apparent activation energies and regression coefficients obtained from six model-free methods for Hassawi rice straw.

	FR		FWO		KAS		STK		K		VY		Average	
Conversion	E kJ mol^−1^	R^2^	E kJ mol^−1^	R^2^	E kJ mol^−1^	R^2^	E kJ mol^−1^	R^2^	E kJ mol^−1^	R^2^	E kJ mol^−1^	R^2^	E kJ mol^−1^	R^2^
0.1	71.0	0.9826	73.2	0.9851	68.5	0.9809	68.7	0.9811	68.5	0.9809	33.0	NA *	63.8	0.9821
0.2	109.4	0.9909	94.7	0.9857	90.4	0.9824	90.7	0.9826	90.4	0.9824	92.7	NA	94.7	0.9848
0.3	133.2	0.9884	113.0	0.9856	109.3	0.9828	109.6	0.9830	109.3	0.9828	104.6	NA	113.2	0.9845
0.4	163.1	0.9847	131.6	0.9846	128.6	0.9821	128.9	0.9822	128.6	0.9821	116.2	NA	132.8	0.9832
0.5	164.6	0.9364	146.9	0.9709	144.6	0.9667	144.9	0.9669	144.6	0.9667	128.7	NA	145.7	0.9615
0.6	150.8	0.9187	150.5	0.9468	148.1	0.9395	148.4	0.9398	148.1	0.9395	140.0	NA	147.6	0.9369
0.7	154.8	0.9176	153.3	0.9183	150.3	0.9070	150.6	0.9075	150.3	0.9070	146.3	NA	150.9	0.9114
0.8	272.0	0.7738	188.6	0.8671	186.6	0.8521	186.9	0.8527	186.6	0.8521	195.0	NA	202.6	0.8395
0.9	147.8	0.9804	221.7	0.9576	220.6	0.9528	221.0	0.9530	220.6	0.9528	164.4	NA	199.3	0.9593
Average	151.9	0.9415	141.5	0.9557	138.5	0.9496	138.9	0.9499	138.5	0.9496	124.5	NA	139.0	0.9493

* NA: R^2^ is not applicable as the Vyazovkin method is a non-linear iso-conversional method that does not rely on linear regression.

**Table 4:** Kinetic parameters for the D3 diffusion model applied to Hassawi rice straw at four heating rates.

	20 K min^−1^			40 K min^−1^			60 K min^−1^			80 K min^−1^			Average
	*E_a_* (kJ/mol)	Ln (A_0_)	R^2^	*E_a_* (kJ/mol)	Ln (A_0_)	R^2^	*E_a_* (kJ/mol)	Ln (A_0_)	R^2^	*E_a_* (kJ/mol)	Ln (A_0_)	R^2^	*E_a_* (kJ/mol)
Reaction mechanism 1 step reaction	72	13.7	0.996	74.1	14.5	0.9985	74.3	15.2	0.9988	72	15.2	0.9997	73.1
Reaction mechanism 2 step reaction	105.2	6.9	0.9988	128.3	3.5	0.9982	132.1	20	0.9981	143.4	2.1	0.9953	127.3
Reaction mechanism 3 step reaction	65.3	15.8	0.9964	57.6	17.8	0.9877	52.7	19	0.9924	46.6	20.2	0.9887	55.6
Average													101.6

**Table 5:** Pre-exponential factors and thermodynamic parameters (ΔH, ΔG, ΔS) for Hassawi rice straw combustion.

	**FR**					**FWO**				
**α**	**R^2^**	**A_0_, min^−1^**	**ΔH (kJ mol^−1^)**	**ΔG (kJ mol^−1^)**	**ΔS (kJ mol^−1^ K^−1^)**	**R^2^**	**A_0_, min^−1^**	**ΔH (kJ mol^−1^)**	**ΔG (kJ mol^−1^)**	**ΔS (kJ mol^−1^ K^−1^)**
0.1	0.9826	3.60 × 10^4^	66.7	173.9	−0.2047	0.9851	4.99 × 10^6^	68.8	154.5	−0.1637
0.2	0.9909	1.85 × 10^8^	104.4	184.8	−0.1348	0.9857	5.98 × 10^8^	89.7	164.3	−0.1250
0.3	0.9884	2.90 × 10^10^	128.3	183.5	−0.0927	0.9856	3.09 × 10^10^	108.0	163.0	−0.0922
0.4	0.9847	1.41 × 10^13^	158.1	182.7	−0.0413	0.9846	1.50 × 10^12^	126.6	162.3	−0.0599
0.5	0.9364	1.54 × 10^13^	159.7	183.9	−0.0406	0.9709	3.42 × 10^13^	142.0	162.2	−0.0339
0.6	0.9187	3.46 × 10^11^	145.8	188.8	−0.0721	0.9468	4.90 × 10^13^	145.6	164.0	−0.0309
0.7	0.9176	7.41 × 10^10^	149.9	200.5	−0.0849	0.9183	2.02 × 10^13^	148.3	171.2	−0.0383
0.8	0.7738	5.72 × 10^18^	265.9	218.7	0.0644	0.8671	1.33 × 10^15^	182.5	186.4	−0.0052
0.9	0.9804	2.78 × 10^9^	141.7	225.2	−0.1139	0.9576	7.50 × 10^16^	215.6	194.8	0.0283
	**KAS**					**STK**				
**α**	**R^2^**	**A_0_, min^−1^**	**ΔH (kJ mol^−1^)**	**ΔG (kJ mol^−1^)**	**ΔS (kJ mol^−1^ K^−1^)**	**R^2^**	**A_0_, min^−1^**	**ΔH (kJ mol^−1^)**	**ΔG (kJ mol^−1^)**	**ΔS (kJ mol^−1^ K^−1^)**
0.1	0.9809	1.50 × 10^4^	64.1	175.1	−0.2120	0.9811	2.69 × 10^4^	64.4	172.8	−0.2071
0.2	0.9824	2.64 × 10^6^	85.5	186.9	−0.1701	0.9826	4.75 × 10^6^	85.8	184.3	−0.1652
0.3	0.9828	1.83 × 10^8^	104.3	184.7	−0.1349	0.9830	3.30 × 10^8^	104.6	182.1	−0.1299
0.4	0.9821	1.16 × 10^10^	123.6	183.5	−0.1004	0.9822	2.09 × 10^10^	123.9	180.9	−0.0955
0.5	0.9667	3.18 × 10^11^	139.6	183.1	−0.0728	0.9669	5.75 × 10^11^	139.9	180.4	−0.0679
0.6	0.9395	4.50 × 10^11^	143.1	184.8	−0.0699	0.9398	8.18 × 10^11^	143.4	182.1	−0.0650
0.7	0.9070	1.69 × 10^11^	145.3	191.9	−0.0781	0.9075	3.09 × 10^11^	145.7	189.2	−0.0731
0.8	0.8521	1.45 × 10^13^	180.5	211.9	−0.0428	0.8527	2.66 × 10^13^	180.8	208.5	−0.0377
0.9	0.9528	1.02 × 10^15^	214.5	220.0	−0.0075	0.9530	1.87 × 10^15^	214.9	216.6	−0.0024
	**K**									
**α**	**R^2^**	**A_0_, min^−1^**	**ΔH (kJ mol^−1^)**	**ΔG (kJ mol^−1^)**	**ΔS (kJ mol^−1^ K^−1^)**					
0.1	0.9809	1.26 × 10^7^	64.1	145.8	−0.1560					
0.2	0.9824	5.14 × 10^8^	85.5	160.8	−0.1263					
0.3	0.9828	1.45 × 10^10^	104.3	163.0	−0.0985					
0.4	0.9821	4.71 × 10^11^	123.6	165.1	−0.0695					
0.5	0.9667	7.46 × 10^12^	139.6	167.4	−0.0466					
0.6	0.9395	6.50 × 10^12^	143.1	171.6	−0.0477					
0.7	0.9070	1.55 × 10^12^	145.3	180.9	−0.0597					
0.8	0.8521	8.40 × 10^13^	180.5	201.1	−0.0282					
0.9	0.9528	3.54 × 10^15^	214.5	212.4	0.0029					

## Data Availability

The original contributions presented in this study are included in the article/Supplementary Material. Further inquiries can be directed to the corresponding author.

## Supplementary Materials

The following supporting information can be downloaded at: https://www.mdpi.com/article/10.3390/polym18131642/s1, Figure S1: Particle size distribution and retained mass fractions of the milled HRS obtained via mechanical sieving, demonstrating a bulk average particle size of ~0.285 mm. Table S1: Summary of model-free and model-fitting methods used for the kinetic analysis of PS combustion, including their corresponding equations and regression plots. Taken from Mousa et. al, 2025 [17]. Table S2: Fifteen of solid–state reaction mechanism. Taken from Mousa et. al, 2025 [17]. Table S3: Thermodynamic parameters, including ΔH, ΔG and ΔS for PS combustion were calculated using the following equation: [17]. Table S4: Kinetic parameters obtained by the CR method for HRS combustion at four heating rates.
