# Supplementary material for: Assessing Hassawi Rice Straw as a Solid Biofuel: High Heating Rate Combustion Behaviour, Kinetics, and Thermodynamic Analysis
Source: Polymers (Basel). 2026 Jul 1;18(13):1642. doi: 10.3390/polym18131642 (PMC13364148; doi:10.3390/polym18131642)
Supplement: Supplementary file 1 [file polymers-18-01642-s001.zip › polymers-4351732-supplementary.pdf]

polymers

MDPI

Supplementary Materials for

Assessing Hassawi Rice Straw as a Solid Biofuel: High Heating Rate Combustion Behaviour, Kinetics, and Thermodynamic Analysis

Mohamed Anwar Ismail [1], Ibrahim Dubdub [2,*], Suleiman Mousa [2] and Abdulrahman Almithn [2]

[1] Mechanical Engineering Department, College of Engineering, King Faisal University, P.O. Box 380, Al-Ahsa 31982, Saudi Arabia
[2] Chemical Engineering Department, College of Engineering, King Faisal University, P.O. Box 380, Al-Ahsa 31982, Saudi Arabia
* Correspondence: idubdub@kfu.edu.sa; Tel.: +966-13-5896989

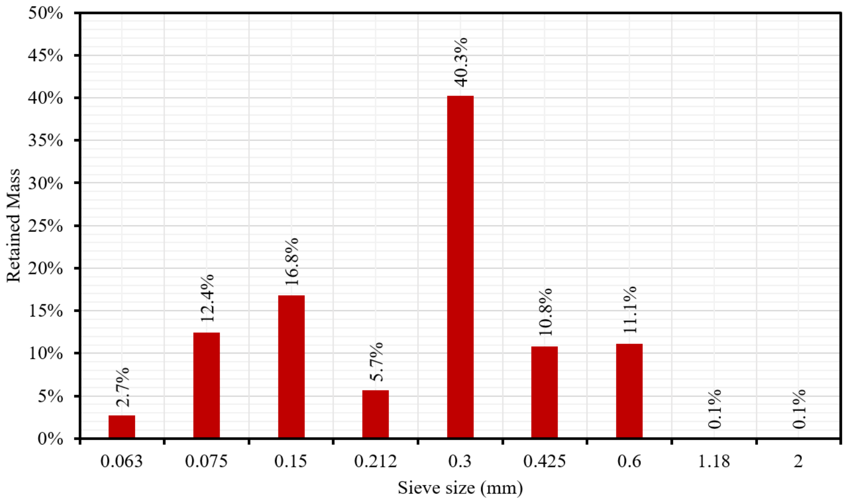

Figure S1. Particle size distribution and retained mass fractions of the milled HRS obtained via mechanical sieving, demonstrating a bulk average particle size of ~0.285 mm.

List of Symbols

α = Degree of conversion
β = Heating rate (K min^{-1})
T = Absolute temperature (K)
T_m = Maximum temperature (K)
T_p = Peak temperature (K)
E_a = Apparent activation energy (kJ mol^{-1})
A_0 = Pre-exponential factor (min^{-1})
R = Universal gas constant (8.314 J K^{-1} mol^{-1})
$f(\alpha)$ = Differential solid-state reaction model
$g(\alpha)$ = Integral solid-state reaction model
ΔH = Enthalpy change (kJ mol^{-1})
ΔG = Gibbs free energy change (kJ mol^{-1})
ΔS = Entropy change (kJ mol^{-1} K^{-1})
k_B = Boltzmann constant (1.381 × 10^{-23} J K^{-1})
h = Planck constant (6.626 × 10^{-34} J s)

Table S1. Summary of model-free and model-fitting methods used for the kinetic analysis of PS combustion, including their corresponding equations and regression plots. Taken from Mousa et. al, 2025 [17].

model-free methods		
**Method**	**Formula**	**Plot**
FR	$ln\left(\beta\frac{d\alpha}{dT}\right) = ln[A_o f(\alpha)] - \frac{E_a}{RT}$ (S4)	$ln\left(\beta\frac{d\alpha}{dT}\right) vs. \frac{1}{T}$
FWO	$ln(\beta) = ln\frac{A_o E_a}{Rg(\alpha)} - 5.331 - 1.052\frac{E_a}{RT}$ (S5)	$ln(\beta)\ vs.\ \frac{1}{T}$
KAS	$ln\left(\frac{\beta}{T^2}\right) = ln\frac{A_o R}{E_a\, g(\alpha)} - \frac{E_a}{RT}$ (S6)	$ln\left(\frac{\beta}{T^2}\right) vs.\ \frac{1}{T}$
STK	$ln\frac{\beta}{T^{1.92}} = ln\left(\frac{A_0 E_a}{Rg(\alpha)}\right) - 1.0008\frac{E_a}{RT}$ (S7)	$ln\frac{\beta}{T^{1.92}}\ vs.\ \frac{1}{T}$
K	$\ln(\frac{\beta}{T_m^2}) = ln\left(\frac{A_0 R}{E_a}\right) - \frac{E_a}{RT}$ (S8)	$\ln(\frac{\beta}{T_m^2})\ vs.\ \frac{1}{T}$
VY	$\Phi(E_\alpha) = \sum_{i=1}^{n}\sum_{j\neq i}^{n}\frac{J[E_\alpha, T_i(t_\alpha)]}{J[E_\alpha, T_j(t_\alpha)]} = 0$ (S9)	minimizing the function $\Phi(E_\alpha)$
model-fitting methods		
**Method**	**Formula**	**Plot**
CR	$ln\left[\frac{g(\alpha)}{T^2}\right] = ln\left[\frac{A_o R}{\beta E_a}\right] - \frac{E}{RT}$ (S10)	$ln\left[\frac{g(\alpha)}{T^2}\right] vs.\frac{1}{T}$

Table S2. Fifteen of solid–state reaction mechanism. Taken from Mousa et. al, 2025 [17].

**Reaction mechanism**	**Code**	$\boldsymbol{f(\alpha)}$	$\boldsymbol{g(\alpha)}$
Reaction order models-1st order	F1	1-α	$-\ln(1-\alpha)$
Reaction order models-2ndorder	F2	$(1-\alpha)^2$	$(1-\alpha)^{-1}-1$
Reaction order models-3rd order	F3	$(1-\alpha)^3$	$[(1-\alpha)^{-2}-1]/2$
Diffusion model-1 dimension	D1	$1/2\alpha^{-1}$	α^2
Diffusion model-2-dimension	D2	$[-ln(1-\alpha)]^{-1}$	$(1-\alpha)\ln(1-\alpha)+\alpha$
Diffusion model-3-dimension	D3	$3/2[1-(1-\alpha)^{1/3}]^{-1}$	$[1-(1-\alpha)^{1/3}]^2$
Nucleation models-2 dimension	A2	$2(1-\alpha)[-\ln(1-\alpha)]^{1/2}$	$[-\ln(1-\alpha)]^{1/2}$
Nucleation models-3-dimension	A3	$3(1-\alpha)[-\ln(1-\alpha)]^{1/3}$	$[-\ln(1-\alpha)]^{1/3}$
Nucleation models-4-dimension	A4	$4(1-\alpha)[-\ln(1-\alpha)]^{1/4}$	$[-\ln(1-\alpha)]^{1/4}$
Geometrical contraction models-1-dimension	R1	1	α
Geometrical contraction models - sphere	R2	$2(1-\alpha)^{1/2}$	1-$(1-\alpha)^{1/2}$
Geometrical contraction models - cylinder	R3	$3(1-\alpha)^{1/3}$	1-$(1-\alpha)^{1/3}$
Power law	P2	$2\alpha^{1/2}$	$\alpha^{1/2}$
Power law	P3	$3\alpha^{2/3}$	$\alpha^{1/3}$
Power law	P4	$4\alpha^{3/4}$	$\alpha^{1/4}$

Table S3: Thermodynamic parameters, including ΔH, ΔG and ΔS for PS combustion were calculated using the following equation: [17].

$H = E_a - R\,T_p$	(S11)
$\Delta G = E_a + R\,T_p l\,n\left(\frac{k_B\,T_p}{h\,A_0}\right)$	(S12)
$\Delta S = \frac{\Delta H - \Delta G}{T_p}$	(S13)

where T_p represents the maximum temperature (K), k_B is the Boltzmann constant (1.381×10^{-23} J·K⁻¹), h is the Planck constant (6.626×10^{-34} J·s), and A_o is the pre-exponential factor, (min⁻¹).

Table S4. Kinetic parameters obtained by the CR method for HRS combustion at four heating rates.

Reaction mechanism 1 step reaction	Code	20 K min^{-1}			40 K min^{-1}			60 K min^{-1}			80 K min^{-1}		
		E_a (kJ/mol)	Ln (A_0)	R^2	Ea (kJ/mol)	Ln (A_0)	R^2	Ea (kJ/mol)	Ln (A_0)	R^2	E_a (kJ/mol)	Ln (A_0)	R^2
Reaction order models-First order	F1	32.6	18.0	0.9950	33.6	18.8	0.9982	33.7	19.4	0.9985	32.6	19.6	0.9996
Reaction order models-Second order	F2	35.1	17.4	0.9957	36.1	18.2	0.9985	36.6	18.7	0.9988	35.1	18.9	0.9995
Reaction order models-Third order	F3	37.6	16.8	0.9962	38.7	17.6	0.9988	39.7	18.0	0.9991	37.6	18.2	0.9994
Diffusion models-One dimension	D1	68.8	12.3	0.9956	70.9	13.1	0.9983	70.5	13.9	0.9985	68.8	13.9	0.9997
Diffusion models-Two dimension	D2	70.4	12.6	0.9958	72.5	13.4	0.9984	72.4	14.2	0.9986	70.4	14.2	0.9997
Diffusion models-Three dimension	D3	72.0	13.7	0.9960	74.1	14.5	0.9985	74.3	15.2	0.9988	72.0	15.2	0.9997
Diffusion models-Four dimension	D4	70.9	13.9	0.9959	73.0	14.7	0.9984	73.0	15.5	0.9987	70.9	15.5	0.9997
Nucleation models-Two dimension	A2	12.2	20.8	0.9907	12.5	21.6	0.9965	12.4	22.2	0.9970	12.2	22.5	0.9993
Nucleation models-Three-dimension	A3	5.3	21.3	0.9780	5.5	22.1	0.9915	5.3	22.6	0.9922	5.3	22.9	0.9985
Nucleation models Fourth dimension	A4	1.9	20.9	0.9088	2.0	21.7	0.9620	1.8	22.1	0.9588	1.9	22.5	0.9935
Geometrical contraction models- One dimension phase boundary	R1	30.3	18.5	0.9943	31.2	19.3	0.9978	30.8	20.0	0.9980	30.3	20.2	0.9996
Geometrical contraction models -Contracting sphere	R2	31.4	18.9	0.9947	32.4	19.7	0.9980	32.2	20.4	0.9983	31.4	20.6	0.9996
Geometrical contraction models- Contracting cylinder	R3	31.8	19.3	0.9948	32.8	20.1	0.9980	32.7	20.7	0.9983	31.8	20.9	0.9996
Power law models	P2	11.0	21.1	0.9887	11.3	21.8	0.9955	11.0	22.4	0.9959	11.0	22.7	0.9993
Power law models	P3	4.6	21.4	0.9703	4.7	22.1	0.9877	4.4	22.6	0.9877	4.6	23.0	0.9981
Power law models	P4	1.3	20.7	0.8287	1.4	21.5	0.9204	1.0	21.8	0.8854	1.3	22.3	0.9855

Reaction mechanism 2 step reaction	Code	20 K min^{-1}			40 K min^{-1}			60 K min^{-1}			80 K min^{-1}		
		E_a (kJ/mol)	Ln (A_0)	R^2	Ea (kJ/mol)	Ln (A_0)	R^2	Ea (kJ/mol)	Ln (A_0)	R^2	E_a (kJ/mol)	Ln (A_0)	R^2
Reaction order models-First order	F1	54.1	13.9	0.9991	66.8	12.4	0.9984	68.5	20.5	0.9984	74.3	12.2	0.9957
Reaction order models-Second order	F2	75.6	9.4	0.9998	92.6	7.2	0.9992	94.0	11.8	0.9992	100.3	7.0	0.9977
Reaction order models-Third order	F3	101.1	4.1	0.9997	123.1	0.9	0.9992	123.9	0.2	0.9992	130.6	0.9	0.9981
Diffusion models-One dimension	D1	82.7	9.5	0.9975	101.3	6.9	0.9966	105.3	23.8	0.9966	115.8	5.5	0.9924
Diffusion models-Two dimension	D2	93.1	7.9	0.9982	113.8	5.0	0.9974	117.7	22.1	0.9974	128.7	3.6	0.9939
Diffusion models-Three dimension	D3	105.2	6.9	0.9988	128.3	3.5	0.9982	132.1	20.0	0.9981	143.4	2.1	0.9953
Diffusion models-Four dimension	D4	97.1	8.6	0.9985	118.6	5.5	0.9977	122.5	22.5	0.9977	133.6	4.1	0.9944
Nucleation models-Two dimension	A2	22.2	19.3	0.9985	28.5	19.1	0.9978	29.2	23.6	0.9977	32.1	19.4	0.9941
Nucleation models-Three-dimension	A3	11.6	20.8	0.9975	15.7	21.0	0.9967	16.2	24.3	0.9966	18.0	21.5	0.9916
Nucleation models Fourth dimension	A4	6.2	21.2	0.9950	9.3	21.7	0.9946	9.6	24.4	0.9944	11.0	22.4	0.9872
Geometrical contraction models-One dimension phase boundary	R1	36.5	17.4	0.9967	45.7	16.6	0.9958	47.6	25.5	0.9958	52.9	16.3	0.9908
Geometrical contraction models -Contracting sphere	R2	44.8	16.4	0.9982	55.6	15.3	0.9974	57.5	24.2	0.9974	63.0	15.1	0.9937
Geometrical contraction models- Contracting cylinder	R3	47.8	16.2	0.9985	59.2	15.0	0.9978	61.0	23.7	0.9978	66.7	14.8	0.9945
Power law models	P2	13.4	20.7	0.9937	17.9	20.9	0.9930	18.8	25.8	0.9931	21.4	21.2	0.9857
Power law models	P3	5.7	21.3	0.9840	8.7	21.9	0.9864	9.2	25.5	0.9869	10.9	22.5	0.9753
Power law models	P4	1.9	21.0	0.9171	4.0	22.0	0.9645	4.4	24.9	0.9675	5.6	22.8	0.9486

Reaction mechanism 3 step reaction	Code	20 K min^{-1}			40 K min^{-1}			60 K min^{-1}			80 K min^{-1}		
		E_a (kJ/mol)	Ln (A_0)	R^2	E_a (kJ/mol)	Ln (A_0)	R^2	E_a (kJ/mol)	Ln (A_0)	R^2	E_a (kJ/mol)	Ln (A_0)	R^2
Reaction order models-First order	F1	44.7	16.7	0.9935	37.3	18.5	0.9845	31.8	19.8	0.9901	26.9	20.7	0.9839
Reaction order models-Second order	F2	127.2	3.0	0.9907	101.9	7.8	0.9872	80.9	11.7	0.9921	67.1	14.0	0.9868
Reaction order models-Third order	F3	238.1	-16.0	0.9899	188.5	-7.1	0.9877	146.2	0.3	0.9924	120.3	4.6	0.9873
Diffusion models-One dimension	D1	19.7	21.0	0.9988	19.1	21.8	0.9790	20.4	22.1	0.9875	18.9	22.6	0.9837
Diffusion models-Two dimension	D2	35.8	19.2	0.9982	33.2	20.3	0.9851	32.8	20.9	0.9909	29.8	21.5	0.9872
Diffusion models-Three dimension	D3	65.3	15.8	0.9964	57.6	17.8	0.9877	52.7	19.0	0.9924	46.6	20.2	0.9887
Diffusion models-Four dimension	D4	45.1	19.2	0.9975	41.0	20.6	0.9864	39.2	21.3	0.9916	35.3	22.1	0.9879
Nucleation models-Two dimension	A2	16.2	20.7	0.9879	12.5	21.8	0.9666	9.8	22.5	0.9746	7.4	22.9	0.9501
Nucleation models-Three-dimension	A3	6.7	21.5	0.9701	4.3	22.2	0.8838	2.5	22.4	0.8468	1.0	22.0	0.4158
Nucleation models Fourth dimension	A4	2.0	21.2	0.8361	0.1	19.5	0.0135	-1.2	#NUM!	0.6997	-2.3	#NUM!	0.8829
Geometrical contraction models-One dimension phase boundary	R1	3.7	21.8	0.9926	3.4	22.5	0.8567	4.1	23.0	0.9267	3.4	23.2	0.8895
Geometrical contraction models -Contracting sphere	R2	19.3	21.2	0.9952	16.7	22.2	0.9759	15.4	22.8	0.9844	13.2	23.3	0.9756
Geometrical contraction models- Contracting cylinder	R3	26.5	20.6	0.9946	22.7	21.8	0.9804	20.3	22.6	0.9873	17.3	23.2	0.9798
Power law models	P2	-4.3	#NUM!	0.9988	-4.4	#NUM!	0.9756	-4.1	#NUM!	0.9803	-4.3	#NUM!	0.9809
Power law models	P3	-6.9	#NUM!	0.9998	-7.0	#NUM!	0.9956	-6.8	#NUM!	0.9968	-6.9	#NUM!	0.9967
Power law models	P4	-8.3	#NUM!	0.9999	-8.3	#NUM!	0.9982	-8.1	#NUM!	0.9987	-8.2	#NUM!	0.9987
